# Why the Better Element of the Dental Profession Does Not Approve of Advertising

**Published:** 1900-02

**Authors:** E. Ballard Loege


ARTICLE VIII.

### WHY THE BETTER ELEMENT OF THE DENTAL PROFESSION DOES NOT APPROVE OF ADVERTISING.

E. BALLARD LOEGE, D. D. S.

It is a misfortune that in dentistry, as in other professions, there are two classes of practitioners, viz. : the non-advertising or professional, and the advertising or unprofessional.

These may be classed as the true and false elements of the profession. Just as in medicine we have the quack and in law the shyster, so in dentistry we are menaced by the advertiser. Now it is a fact that the popular mind does not understand why it is not ethical and right that a dentist should advertise in the public prints. In the commercial world printers' ink and aggressiveness are right and proper; but, in the profession of dentistry, to be over zealous in proclaiming one's self is not only immodest, egotistic and undignified, but is synonymous with charlatanism. You agree, perhaps, that a young man just starting in his professional career would starve to death if he did not resort to advertising as a means of putting himself before the public. Fortunately, such is not the case. There are other and better methods of winning public favor than by the methods employed by a class of practitioners herein dealt with.

A man is permitted by the ethics of dentistry to insert in the newspaper his business card, provided he confines himself to his name, his business and his address, but just as soon as he goes farther than this he is in danger of compromising himself, if not of committing professional suicide. It may be that considerable patience may have to be exercised on the part of the young dental practitioner, as in all professions it is exceptional for one to escape the so-called starvation period. But when the facts are made clear I am sure you will agree with me that a dentist can not afford to do an advertising business; surely he can not if he loves the profession. The truth is a man can not advertise so that it will result in financial gain unless he resorts to exaggeration of his capabilities, or make it appear that he can do for his patient what his brother practitioner cannot do.

This is taking undue advantage, and the man who thus plants himself before the public, the man who would be this extraordinary phenomenon, degrades himself and his calling and is an outcast so long as he persists in those methods. It is absurd to imagine that a man who thus poses before the public, will render a service to his patient superior to that of the more modest, more conscientious practitioner, who is wise and honest enough to acknowledge his own limitations. If, for example, it is possible for a pretender to extract a tooth without pain and with anesthesia, local or general, is done many times, is it not quite as reasonable to expect a reputable dentist to do so, and would you not expect such an one to be just as thorough in his operation, and to take just as strict measures to prevent infecting his patient with an unclean instrument or an unsterilized solution ? I mention this latter because two cases of necrosis of the jaw have been brought to my attention, the unfortunates having been infected at institutions of quackery. There seems to be a popular impression that the non-advertising dentist is not "on to the trick" of doing certain operations which are done successfully (?) by advertisers; but permit me to assure you that the most progressive dentists, and those with whom it is most safe to entrust your welfare, are the men who love their calling too much to jeopardize their patient's health for the sake of the dollar. And it is most absurd to think that just because a man does not inflate himself like a toad in a marsh, and make a loud noise he cannot be capable of good work. It is much to be deplored that so many intelligent persons will allow themselves to be duped by such advertisements, as are seen in the daily papers and on billboards, telephone poles and fences. I believe it was Barnum who said "the American people like to be humbugged," and it seems as if this were pretty true when we see how many persons go to the advertising "parlors" for their dentistry. It is true, however, that they do not as a rule continue to patronize the same institution. Advertisers are dependent largely upon a transient class of practice. Oftentimes these institutions of quackery are conducted by men who have had no professional training whatever, but who thoroughly understand the principles of business. Such a man will employ dentists on a salary and the prime idea of this employe is to turn out work, and with less regard to the quality than the quantity of operations performed. I am personally acquainted with a dentist in this city who left a place of the above description—a place well known for its advertising propensity, who told me that while receiving a fair salary he could not be dishonest enough with his patient to please his employer. This young man is now conducting an honorable private practice.

To conclude—respected reader—remember that the so-called cheap is very apt to be expensive in the end, and that when dental operations are in question, the best service is always the cheapest. Therefore, if you are wise, entrust yourself to a man who is willing to win and maintain his reputation by the character of his work, and one who does not feel the need of obscuring his identity by the name "dental parlors."—*Information.*

---

### Communicated.

